# Eco-friendly micellar HPLC for metformin and bisoprolol analysis in diverse matrices with a green and blueness perspective on drug purity and safety

**DOI:** 10.1038/s41598-025-22712-w

**Published:** 2025-10-30

**Authors:** Naglaa Abdel Sattar Kabil, Magda Mohamed El-Henawee, Mona Abd Elnasser Labib, Eman Darweish

**Affiliations:** 1 Pharmaceutical Analytical Chemistry, Faculty of Pharmacy, Zagazig University, Zagazig, 44519 Egypt; 2 Pharmaceutical Chemistry Department, Faculty of Pharmacy, Egyptian Russian University, Badr City, Cairo, 11829 Egypt

**Keywords:** Metformin, Bisoprolol, Impurities, Green assessment and blueness, Diseases, Chemistry

## Abstract

Green analytical chemistry is an innovative approach to making analytical experiments more environmentally friendly, safer for humans, and less toxic to the environment by utilizing eco-friendly reagents and chemicals. Diabetics are twice as likely to experience hypertension compared to nondiabetics. Individuals with both conditions face a significantly increased risk of early microvascular and macrovascular complications. Regular monitoring helps ensure the drug concentration remains within the therapeutic range, maximizing its effectiveness. Herein, a high-performance liquid chromatographic (HPLC) method is provided to evaluate and isolate the studied drugs in the presence of their toxic impurities. The study involves the simultaneous administration of two crucial drugs: the antidiabetic metformin and the antihypertensive bisoprolol. We have investigated their pure form in spiked human plasma, as well as their interaction while their possible toxic impurities are present as melamine and cyanoguanidine. The optimal conditions were achieved using a mobile phase consisting of 0.1 M SDS, 0.1% ortho-phosphoric acid in water, and 10% isopropanol at a flow rate of 1.0 mL/min. The pH was adjusted to 5.0 using triethylamine, and the column temperature was maintained at 25 °C on a symmetry C18 column (Kinetex 1.7µ C18 100A, 2.1-mm × 50-mm), with detection at 230 nm. Following the International Conference of Harmonization guideline, the method was successfully validated within the linearity range of 1–25 µg/mL for metformin and bisoprolol. Notably, the LOQ was determined to be 0.92 µg/mL for metformin and 0.54 µg/mL for bisoprolol. The established method epitomizes accuracy, cost-effectiveness, selectivity, and reproducibility. The evaluation of the suggested approaches’ level of greenness was meticulously conducted using a variety of assessments, including the Analytical eco-scale system, the Modified Green Analytical Procedure Index (MoGAPI), The Analytical Green Star Area (AGSA), Blueness evaluation using BAGI tools, Carbon footprint (CaFRI), Click Analytical Chemistry Index (CACI) and White analytical chemistry (WAC).

**Supplementary Information:**

The online version contains supplementary material available at 10.1038/s41598-025-22712-w.

## Introduction

Green chemistry is an ambitious effort aimed at developing reliable, precise, and successful approaches without causing harm to people. To effectively protect the environment, it is crucial to utilize green solvents, catalysts and minimize waste generation. Furthermore, we must prioritize the preparation of samples and ensure optimal energy usage in our tools^1^. The prevalence of hypertension and diabetes is on the rise. Diabetics are more likely to experience hypertension compared to nondiabetics, which plays a significant role in causing death and disability^2^. Most individuals with diabetes and hypertension need to take two or more medications at the same time to effectively control their high blood pressure and blood sugar^3^. People metabolize and respond to medications differently. Even without significant changes, Therapeutic Drug Monitoring (TDM) helps ensure the drug is at the right level for each individual. Consequently, regular TDM and open communication with your healthcare provider is essential for adjusting your medication and managing your health condition effectively^4^. Consequently, the intended drugs were determined by spiking the plasm as a co-administration.

**Fig. 1:**
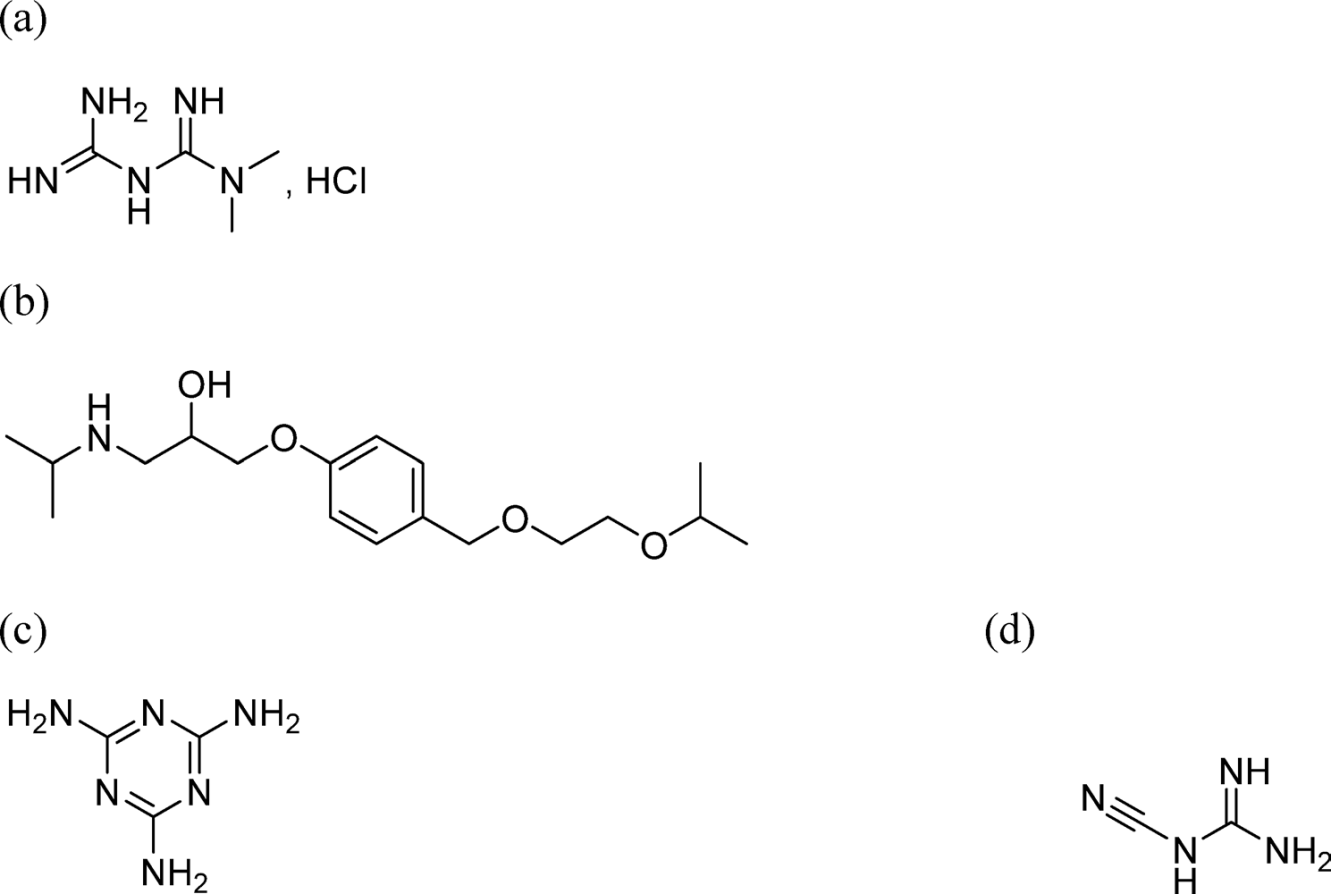
Chemical structure of metformin hydrochloride (**a**), bisoprolol (**b**), melamine (**c**) and cyanoguanidine (**d**).

Metformin(MET), falls under the category of Biguanide, specifically 1,1-dimethyl biguanide hydrochloride^5^ (Fig. 1a). It is prescribed as a medication for managing blood glucose levels in individuals with type II diabetes^6^. It helps to reduce blood glucose levels. Reduced hepatic glucose synthesis, specifically gluconeogenesis, leads to increased insulin sensitivity in the peripheral tissues; this effect is obvious.

Bisoprolol fumarate (BISO) is the name of a synthetic beta1-selective and cardioselective adrenoreceptor blocker, also known as. The chemical name for bisoprolol fumarate is 1-{4-[(2-isopropoxy ethoxy) methyl] phenoxy}-3-(isopropylamino) propan-2-ol fumarate^5^ (Fig. 1b), which is used to treat hypertension, angina pectoris, and arrhythmia^7^.

When beta-blockers such as BISO are co-administered with MET, they can significantly mask the early warning signs of hypoglycemia, such as an irregular heartbeat, which happens when blood sugar levels drop too low. Therefore, accurate monitoring of the plasma concentrations of both drugs in spiked samples is critical. BISO is effective in managing the pathogenetic mechanisms underlying hypertension and metabolic syndrome, while MET enhances carbohydrate and lipid metabolism. Peak plasma concentrations of MET and BISO range from 1.0 to 1.6 mg/L^8^ and 24.3 ± 0.3 ng/mL^9^. Accordingly, closely monitoring the concentration of the two co-administered drugs in spiked plasma was crucial. So, BISO treatment is useful in treating pathogenetic processes in hypertension and metabolic syndrome. MET also improves indicators of carbohydrate and lipid metabolism^10,11^.

MET included considerable impurities such as melamine (MLN) (Fig. 1c) and cyanoguanidine (CGD) (Fig. 1d). In metformin hydrochloride formulations, MLN and CGN impurities typically originate from the synthesis and degradation pathways of the drug’s raw materials. MLN, a triazine compound, is not directly involved in MET synthesis but can appear as a contaminant due to shared manufacturing equipment, raw material impurities, or degradation of triazine-based compounds. Its presence is closely monitored due to its known toxicity. CGN is structurally related to MET and can form as a residual intermediate or degradation product during the synthesis of MET from dimethylamine hydrochloride and dicyandiamide. If the reaction is incomplete or purification is insufficient, traces of CGN may remain. British Pharmacopoeia requires purity checks for two contaminants in MET: CGD (0.02%) and MEL (0.1%)^12^. MLN in medicines or diets might trigger inflammation and renal issues. The great majority of individuals experienced acute renal impairment, nephrolithiasis, and urolithiasis^13^. Animal investigations have indicated that CGD causes sub-oral toxicity and cutaneous discomfort^14^. Moreover, Impurities can interfere with the drug’s intended mechanism of action, reducing its effectiveness. They might bind to the target site, compete with the active ingredient, or even degrade the active compound. Moreover, it can cause adverse effects ranging from mild allergies to severe toxicity. They can also impact the stability of the drug, leading to degradation and potentially harmful byproducts^15^. The International Conference on Harmonization (ICH) has established comprehensive guidelines for the identification, qualification, and control of impurities in pharmaceuticals. These guidelines aim to ensure consistent quality standards across different regions^16^. Consequently, Pharmaceutical companies conduct rigorous risk assessments to understand the potential impact of impurities on the drug’s safety and efficacy. Consequently, detecting the presence of MLN and CGD impurities proved to be extremely beneficial. Few analytical determination techniques were described for the evaluation of MET and BISO as co-administration, utilizing, such as fluorescence spectrophotometry ^17^, liquid chromatography–tandem mass spectrometry method ^18^, which lack green assessment that uses reagents harmful for the environment and expensive. Different methods were found for the analysis of MET impurities as HPLC^19–21^, HILIC^22^, quadruple devisor spectrophotometric method^23^. After thoroughly reviewing the literature, this study presents, for the first time, the simultaneous determination of MET and BISO in spiked human plasma using a high-performance liquid chromatography (HPLC) method in the presence of their respective impurities, MLN and CGN, in addition to various green analytical assessment tools were employed to evaluate the method’s environmental impact. Several analytical techniques are employed to detect and quantify impurities, including chromatography (HPLC, GC), spectroscopy (NMR, IR, UV), and mass spectrometry. The chromatographic approach is chosen because of its numerous advantages, including its precise separation of materials from small sample amounts and distinct substances with similar chemical and physical properties^24^. A further benefit of HPLC over other techniques is its ability to analyze numerous samples simultaneously using the same mobile phase on a single plate, making it a simple, rapid, and affordable procedure. Consequently, the HPLC method is strongly advised^25^. Consequently, the suggested HPLC methodology provides accurate, reliable, and environmentally safe procedures for the first time to determine MET and BISO containing hazardous impurities such as CGD and MLN in pure form and spiked human plasma. Furthermore, comprehensive profile evaluations were established to evaluate the degree of greenness quantitatively for the developed approaches, including the Analytical eco-scale system (penalty point system). The Modified Green Analytical Procedure Index (MoGAPI) provides a visual evaluation of the environmental impact across all steps by using a pentagram-shaped pictogram with colored fields (green, yellow, red). The Analytical Green Star Area (AGSA) uses a circular pictogram divided into 12 sections that each of which is color-coded from red to green. Along with applying Blueness evaluation using BAGI tools, the concept of “Blueness” is introduced. This metric combines analytical flexibility and efficiency, method effectiveness, and greenness. Besides the carbon footprint (CaFRI), the carbon footprints of different products, bodies, and activities are measured internationally to indicate their greenhouse gas intensity, adhering to the principle that only quantifiable data can be effectively managed. Based on the ideas of click chemistry, click analytical chemistry (CAC) is a new method that prioritizes dependability, efficiency, and simplicity. Although click chemistry transformed synthetic methods, CAC seeks to improve chemical analysis by offering a framework for method evaluation from an alternative viewpoint, emphasizing the usefulness and relevance of analytical methods. White analytical chemistry (WAC) states that to protect the environment and aquatic life, it is essential to replace these hazardous organic solvents with safer and more affordable alternatives. Excellent performance is represented by a colored pictogram, middling performance by gray, and inadequate performance or failure to meet the intended standards by black. White is made by combining the colors red, green, and blue (RGB).

## Materials and methods

### Instruments

#### HPLC–UV system

The Agilent 1100 series chromatographic system, which includes a thermostatic column compartment, a micro vacuum degasser, a quaternary pump, and a variable wavelength UV–VIS detector, was used to conduct the HPLC studies. Additionally, sample injection was performed using an Agilent 1100 series auto sampler. Version A.10.01 of Agilent ChemStation software was used for data collection and processing. Ultrasonic device type: Ultrasonic bath EMAG ultrasonic cleaner Emmi-20 HC, Germany. Separation was performed on a Kinetics 1.7μ C18 100A (2.1-mm × 50-mm) column, which was used for the separation process. The detector is UV at λ max = 230 nm. Injection volume: 10 µL. Flow rate: 1 mL /min. Temperature: 25 °C sonication.

### Chemicals

#### Pure sample

Amoun Pharma Company, Cairo, Egypt, graciously supplied pure standards of MET and BISO with verified purity to be 99.5% and 99.5%, respectively. MLN was obtained from Sigma-Aldrich Co. Chemie GmbH, and CGD was obtained from Sigma Aldrich (St. Louis, MO, USA), which are not to exceed 0.1% and 0.02%, respectively.

#### Materials, reagents, and pharmaceutical products

SDS (Himedia Mumbai, India), Methanol (HPLC grade, Merck, Germany), isopropanol, trimethylamine, and ortho-phosphoric acid (Merck, Darmstadt). Fisher HPLC grade, distilled water, and blank human plasma were given from the holding company for biological products and vaccines, Giza, Egypt, and stored at 20 °C. Glucophage SR tablet® (500 mg MET) from MERCK LIMITED-EGYPT company. Concor tablet® (5 mg BISO) from the MERCK company.

### Standard solutions

The standard stock solutions of MET and BISO (1 mg/mL) were prepared separately in methanol; amounts of the stock solution were taken and diluted using the mobile phase to produce a standard working solution of 50 µg/mL of the medications. Six standard solutions at concentration levels of (1, 5, 10, 15, 20, 25 µg/mL) for MET and (1, 5, 10, 15, 20, 25 µg/mL) for BISO were prepared through serial dilutions from the working solution using the mobile phase for the quantitative validation research. A refrigerator was used to hold stock solutions at 2 °C.

## Procedures

### Chromatographic conditions

A symmetry C18 columns (Kinetex 1.7μ C18 100A (2.1-mm × 50-mm)), Detector: (UV at λ max = 230-nm), Injection volume: 10 µL. The chromatographic separation of the substances under investigation was completed using isocratic elution. The elution utilized a mobile phase consisting of 90% (0.1 M SDS and 0.1% ortho-phosphoric acid in water, pH adjusted to 5.0 by triethylamine) and 10% isopropanol, with a flow rate of 1 mL/min. A Millipore with a 0.45 µm pore size was employed as a membrane filter to strain the mobile phase, and it was purged of gases before usage. Temperature: 25 °C at 230 nm, scanning was conducted.

### Creation of calibration graphs

Portions of the standard working solutions were separated and then added to distinct sets of 10 mL volumetric flasks. After that, the mobile phase was added to them to dilute them to concentration levels of (1–25 µg/mL) for MET and (1–25 µg/mL) for BISO. Then the previously described chromatographic conditions were followed. The drugs’ linearity was verified by creating graphs of the integrated peak areas against the concentrations used, followed by the calculation of regression equations.

### Application on the synthetic pharmaceutical formulation

After weighing, combining, and grinding the ten tablets^26^ of each medication in a porcelain mortar, the average weight was determined. A specific amount of powder 10 mg, equivalent to one tablet of both MET and BISO, was entirely transferred to a 100 mL volumetric flask. Subsequently, 80 mL of the diluent (methanol) was added, so the stock solution is 10 mg/100 mL, and the mixture was subjected to 30 min of sonication. The diluent was used to fill the flask up to the mark. A 0.45 µm membrane filter was employed to filter the resulting solution. Then the previously described chromatographic conditions were followed. For the application with impurities, 10 mg taken from MLN and CGN was then entirely transferred to a 100 mL volumetric flask. Subsequently, 80 mL of the diluent (methanol) was added, so the stock solution of impurities is 10 mg/100 mL.

### Application in spiked human plasma

Five centrifugation tubes were used to process the plasma samples. Each sample, consisting of 1 mL of thawed plasma and 1 mL of standard solutions, was mixed with different standard aliquots from the respective solution of each drug. Following this, 3 mL of methanol/water (1/1) was added. The contents of the tubes were then centrifuged for ten minutes at 5000 rpm to ensure complete segregation of proteins. The supernatant layer was separated and filtered with a syringe filter and quantified using the previously mentioned procedures, and the resulting peaks were used to calculate the corresponding concentrations.

## Results and discussion

The pharmaceutical industry and analytical chemistry research are constantly trying to come up with innovative, simple, low-cost, and efficient techniques for the identification and separation of therapeutic compounds. People and the environment may be impacted by the various hazards and drawbacks encountered along the route. Consequently, providing analytical techniques with the same successful results became a global objective. For the first time, the concentrations of MET and BISO in the presence of possible MET-harmful contaminants, such as MLN and CGD, were determined using novel, simple, and environmentally friendly analytical chromatographic procedures.

### Method development and optimization

It was crucial to conduct experiments that could determine the impact of different variables on the HPLC process to maximize its efficiency. The experiment involved testing different mobile phases with varying compositions and polarities. Initially, various stationary phases with varying properties were tested, including a CN column (250 × 4.6 mm, 5 µm) and Zorbax. Columns used include Eclipse C8 (50 × 2.1 mm, 1.9 µm), Poroshell C18 (150 × 4.6 mm, 4 µm), XTerra HPLC RP-C18 (250 × 4.6 mm, 5 µm), Hypersil Gold C18 (50 × 2.1 mm, 1.9 µm) and a symmetry C18 columns (Kinetex 1.7μ C18 100A (2.1-mm × 50-mm)).

A symmetry C18 columns (Kinetex 1.7μ C18 100A (2.1-mm × 50-mm)) demonstrated superior resolution and good peaks for all substances examined. Several mobile phases with varying polarity and compositions were tested, as 0.05 M SDS and water were used, but the separation was not satisfactory. Subsequently, SDS at 0.2 M was tested, but the separation was still inadequate. Column temperature at 25 °C and 40 were not giving the best resolution so ultimately, the optimal mobile phase was determined to be 90% of (0.1 M SDS, 0.1% ortho-phosphoric acid in water, and 10% iso-propanol at a flow rate of 1.0 mL/min, at pH 5, and with a column temperature of 25 °C. Following testing at various wavelengths, it was found that, as shown in Fig. S2, 230 nm had the best baseline and the highest sensitivity. Thus, as indicated by Table 1, satisfactory results were obtained after computing the tailing factor, selectivity, resolution, and system suitability parameters.

**Table 1:** Assay validation parameters of the developed chromatographic methods for determination of MET and BISO.

Method parameters	MET	BISO
Linearity range(µg/mL)	1.0–25.0	1.0–25.0
Linearity equation	y = 11.443 × − 1.588	y = 6.292 × − 0.126
r^2^	0.9999	0.9999
Regression equation parameters		
Slope	11.443	6.292
Intercept	− 1.588	− 0.126
Correlation coefficient (r)	0.9999	0.9999
Accuracy (Mean ± SD)	100.14 ± 1.69	100.17 ± 0.38
Precision		
(± %RSD)a	0.599	0.799
(± %RSD)b	4.396	0.847
LOD (µg/mL)	0.305	0.178
LOQ (µg/mL)	0.924	0.541
Robustness (%RSD)c		
Buffer volume	1.307	1.301
pH	1.363	1.115
Flow rate (mL/min)	0.604	0.557

Repeatability (%RSD a) (n = 3). %RSD using three concentrations within the linear range for each standard repeated three times within the same day.

Intermediate (%RSD b) (n = 3). %RSD using three concentrations within the linear range for each standard repeated three times within three successive days.

(%RSD c) Average of the change in buffer volume (± 2%), pH (± 0.5) and flow rate (± 0.2 mL/min).

#### Toxicity profile of analytical reagents used in HPLC method

- Methanol while commonly used as a solvent, is known to be toxic and flammable; however, it was used in minimal volumes, and its use was justified due to its high extraction efficiency and compatibility with the analytes.
- The mobile phase components—SDS (sodium dodecyl sulfate), ortho-phosphoric acid, isopropanol, and triethylamine—have varying degrees of toxicity:
  - SDS is a biodegradable surfactant, but it can irritate the skin and eyes.
  - Ortho-phosphoric acid is corrosive but was used in diluted form and small amounts.
  - Isopropanol is less toxic compared to acetonitrile and is classified as a greener solvent.
  - Triethylamine, while volatile and odorous, was used at low concentrations and handled in a well-ventilated environment to minimize exposure.

We also performed a greenness assessment using different tools, which provided a quantitative indication of the method’s environmental footprint. The overall scores suggest that the method offers a reasonable balance between analytical performance and environmental safety.

### Method validation

The validation of the analytical procedures was conducted by the ICH guideline^27^ as presented in Table 1.

#### Linearity

**Fig. 2:**
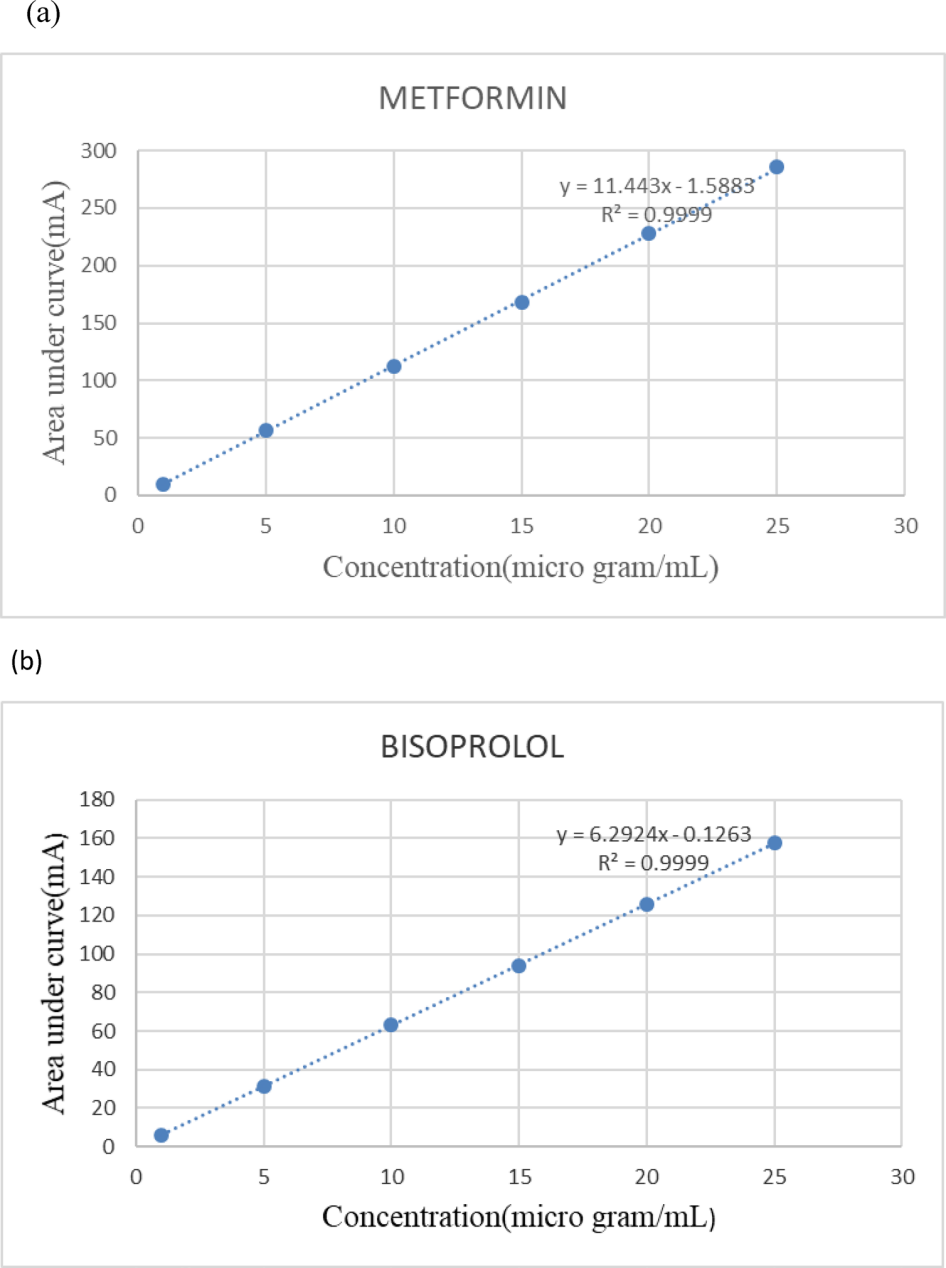
Linearity of the peak area at 230 nm to the corresponding concentration of (**a**) MET (1–25 µg/mL) and (**b**) BISO (1–25 µg/mL) using HPLC method.

The suggested platforms’ linearity was evaluated by measuring the integrated peak area of various concentrations for each MET and BISO under ideal chromatographic conditions (Fig. 2a and b) show the calibration curves that were created between the peak area of each drug and the associated concentration for MET and BISO, respectively, while R2 values were calculated and showed excellent linearity in Table1.

#### Limits of quantification (LOQ) and detection limits (LOD)

The sensitivity of the established approaches was rigorously assessed by computing the LOD and LOQ in strict accordance with ICH recommendations; the recommended method’s acceptable sensitivity was unequivocally verified by exceedingly low values for both LOD and LOQ, where LOQ = 10 × (SD of the response/slope) and LOD = 3.3 × (SD of the response/slope). SD is the intercept’s standard deviation. The findings, as depicted in Table 1, undeniably demonstrate the sufficiency of the approach.

#### Accuracy

For the accuracy investigation, the mean % of MET and BISO was determined using six different concentrations. The results in Table 2 showed that the developed method was valid.

**Table 2:** Results of accuracy for determination of MET and BISO by the proposed HPLC method.

MET			BISO		
Taken (µg/mL)	Found* (µg/mL)	Recovery %	Taken (µg/mL)	Found* (µg/mL)	Recovery %
1.00	1.02	101.27	1.00	0.97	97.38
5.00	5.087	101.73	5.00	5.01	100.26
10.00	9.98	99.82	10.00	10.09	100.95
15.00	14.81	98.71	15.00	14.91	99.41
20.00	20.03	100.15	20.00	19.69	99.81
25.00	25.08	100.32	25.0	25.04	100.17
Mean ± SD	100.14 ± 1.69		Mean ± SD	100.17 ± 0.38	

*Average of three determinations.

#### Precision

The study examines drugs by conducting three repetitions of three concentrations within the same day to evaluate repeatability. The results are estimated using the relative standard deviation (RSD% %), as depicted in Table 1. For assessing intermediate precision, we conducted three separate runs of the same three chosen drug concentrations on different days (inter-day) and calculated the RSD% as depicted in Table 1.

#### Robustness

The robustness evaluation carefully assessed deliberate variations in chromatographic conditions, including the buffer volume (± 2%), pH (± 0.5), and flow rate (± 0.2 mL/min). One parameter changed while the other factors stayed the same; the findings revealed no appreciable differences when using the developed approaches. The computed differences were expressed using the percentage RSD values, which yielded results of less than 2% as in Table 1.

#### System Suitability

In pharmaceutical laboratories, it was once believed that system appropriateness testing was the best way to determine whether a chromatographic system is suitable for a specific analysis, given the crucial nature of result quality. The following parameters were included in the system suitability tests (SST) report: Efficiency (N), Tailing factor (T), Resolution (Rs), retention time, Efficiency (N), Selectivity factor (α), and height equivalent to theoretical plates (HETP) (cm/plate). All the parameters have been calculated, and appropriate values have been obtained and presented in Table 3.

**Table 3:** System suitability parameters of the proposed chromatographic methods for determination of CGN, MET, MLN and BISO.

Parameter	CGN	MET	MLN	BISO
Retention time (t_R_) (min ± 0.2)	1.75	2.36	3.059	3.85
Number of theoretical plates (N)	4015	4365	3912	4898
Resolution (Rs)	4.52	3.12		3.89
Selectivity factor (α)	7.12	6.18		7.89
Tailing factor (T)	0.85	1.16	0.88	1.20
Height equivalent to theoretical plates (HETP) (mm/plate)	0.012	0.011	0.010	0.010

### Application of synthetic pharmaceutical formulation

The proposed procedures were able to determine the medicines MET and BISO in their synthetic pharmaceutical formulation without the interference of any additives or excipients. This was achieved by using 5 different concentrations for each drug. The results, as seen in Table 4, were sufficient and acceptable. HPLC chromatogram in Fig. S1 showing prepared pharmaceutical of MET (10 µg/mL) and BISO (10 µg/mL) with retention times are (2.37) and (3.85) respectively.

**Table 4:** Application of synthetic pharmaceutical formulation for determination of MET and BISO.

	Taken (µg/mL)	Found (µg/mL)	Recovery %	Mean ± SD
MET	2.50	2.56	102.62	100.29 ± 1.62
	5.00	5.01	100.27	
	7.50	7.36	98.13	
	10.00	9.97	99.76	
	12.50	12.58	100.67	
BISO	2.50	2.51	100.69	100.15 ± 1.29
	5.00	5.08	101.63	
	7.50	7.38	98.47	
	10.00	9.91	99.13	
	12.50	12.60	100.80	

### Specificity

To ensure specificity, laboratory-prepared solutions with varying amounts of MET impurities (2.5, 5, 7.5, and 10 µg/mL) and a synthetic pharmaceutical formulation ratio (10:10) were tested in spiked human plasma. Results showed excellent mean percentage recoveries, SD, and %RSD values as in Table 5. Selectivity was shown by the high resolution and separation of MET, BISO, MLN, and CGD as shown in Fig. S3.

**Table 5:** Analysis results of laboratory prepared mixtures containing different percentages of MET impurities (MEL and CGD) by the developed chromatographic method.

	Taken (µg/mL)				HPLC			
	MET	BISO	MLN	CGD	MET	BISO	MLN	CGD
% Recovery	10	10	2.5	2.5	98.38	98.66	102.12	101.87
	10	10	5	5	98.33	98.21	99.10	98.90
	10	10	7.5	7.5	98.43	98.79	99.06	98.92
	10	10	10	10	101.92	101.95	101.40	101.57
Mean					98.27	99.41	100.42	100.32
SD					1.77	1.72	1.58	1.63
%RSD					1.79	1.73	1.57	1.63

### Application in spiked human plasma

The proposed HPLC method demonstrated high sensitivity, as evidenced by the low LOD, enabling accurate quantification of the studied drugs in human plasma. This is supported by the results presented in Table 6. Blank plasma chromatogram shown in Fig. S4a. The chromatogram shown in Fig. S4b demonstrates that the proposed method can be effectively applied in bioequivalence studies for the target drugs and their impurities in spiked human plasma simultaneously without interference from biological fluids’ matrices.

**Table 6:** Application in plasma for determination of MET and BISO.

	Taken (µg/mL)	Found (µg/mL)	Recovery %	Mean ± SD
MET	5.00	4.99	99.88	99.15 ± 1.45
	10.00	9.79	97.99	
	15.00	14.73	98.21	
	20.00	19.66	98.30	
	25.00	25.34	101.38	
BISO	5.00	5.07	101.48	101.24 ± 1.003
	10.00	10.20	102.07	
	15.00	14.94	99.61	
	20.00	20.20	101.03	
	25.00	25.50	102.00	

## Statistical analysis

The reported HPLC method for MET^28^ and BISO^29^ was statistically compared to the obtained results from MET and BISO in their manufactured pharmaceutical formulation using the recommended procedures. The calculated and tabulated t and F values, as shown in Table 7, confirmed that there are no statistical variances between them.

**Table 7:** Statistical analysis of the adopted methods and the reported method of MET and BISO^20,21^ **.

Parameter	Proposed HPLC method		Reported HPLC method	
	MET	BISO	MET	BISO
Mean	99.92	100.17	100.61	100.19
SD	1.40	0.38	1.91	0.81
n	5	5	5	3
Variance	1.97	0.14	3.67	0.14
Student’s *t*-test b	0.65(2.31)*	1.43 (2.45)*	–	–
F value b	1.86(6.39)*	1.05 (19.25)*	–	–

**The reported method is an HPLC method where 233 nm were used for detection of MET with mobile phase consisted of methanol–water (30:70 v/v), and for BISO was detected at 225 nm with mobile phase was a mixture of phosphate buffer (pH = 3.5) and acetonitrile (70:30).

*The values between parenthesis are the theoretical values for t and F at *P* = 0.05.

## Assessing the environmental impact of different analytical procedures

Assessing the environmental impact of different analytical procedures in terms of their adherence to green chemistry principles is crucial, rather than depending on the authors’ subjective viewpoints or assumptions**.** In order to evaluate the environmental friendliness of the proposed methods, we utilized Eco-scale, MoGAPI, AGSA, BAGI, CaFRI, CACI and WAC metrics.

### Analytical eco-scale system (penalty point)

This semi-quantitative evaluation technique uses penalty points for each item on the scale^30^. Penalty points were allocated to criteria that may impact the analytical process, including instrumentation, energy consumption, solvents used, and waste. To calculate the method’s base value, remove the sum of created and predictable dangers from 100 as this equation (Eco-Scale Score) = 100 − ∑ Penalty Points = 100 − 19 = 81. After applying these criteria to the proposed methods, the eco-score value was 81 for the HPLC method as presented in Table 8 so it is an excellent green method.

**Table 8:** Greenness assessment of the proposed chromatographic methods by analytical eco-scale.

Eco-scale assessment	Proposed HPLC method
Reagent	
ISO propanol	4
Orto-phosphoric acid	2
Water	0
Sodium dodecyl sulfate	6
Energy consumption	1
Occupational Hazard	0
Waste amount	3
Waste treatment	3
Total PPs	19
Analytical eco-scale total score	81
Comment	Excellent green analysis

### Green assessment of the developed methods

#### Modified Green analytical procedure index (MoGAPI)

**Fig. 3:**
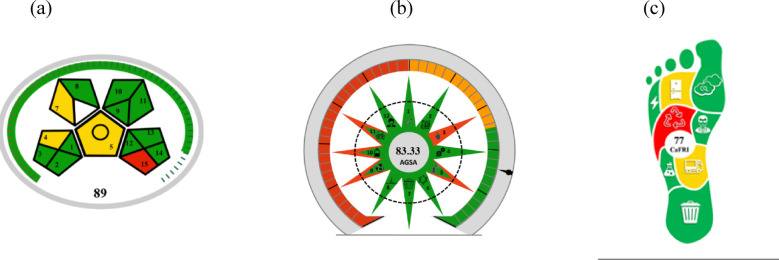
Green assessment of the developed method by MoGAPI (**a**), AGSA (**b**), and CaFRI (**c**) pictograms.

It is similar to the green analytical procedure index (GAPI) but MoGAPI is better as the GAPI metric does not provide a total score that would allow for technique comparison. To overcome the shortcomings of the current GAPI metric, a modified GAPI tool (MoGAPI) and software have been created and implemented in this study. The tool that is being given provides a more accurate measure of greenness, while the software streamlines and speeds up its use, the tool that is being given provides a more accurate evaluation of greenness^31^. The GAPI evaluation was performed according to MoGAPI (available at bit.ly/MoGAPI), which consists of 15 parameters represented by five pentacle shapes. It is designed to quantify and evaluate the environmental impact of each stage of an analytical method, including sample preparation, solvent and reagent health hazards, instrumentation, and waste management. A color-coded system is used, with green indicating low environmental impact, yellow indicating medium impact, and red indicating high impact. Red zones were observed in solvent use (methanol, TEA), while green zones were noted in energy and instrument type (HPLC with UV detection). The MoGAPI pictogram in the developed method included a valuation score of 89, most sectors showed green status as shown in Fig. 3a.

#### The analytical green star area (AGSA)

It is similar to the Analytical GREEnness metric, but it lacks an equivalent statistic in GC, is less immune to user bias, and does not categorize analytical methods according to overall scores. The Analytical Green Star Area (AGSA) offers a thorough, integrated grading system and a visually intuitive method of evaluation to fill these gaps, which assesses that the proposed techniques adhere to the environmental green principle. keeping by the GAC’s 12 Principles. Additionally, AGSA is an expansion of a comparable statistic from GC^32^. The AGREE pictograms for the proposed reveal according to this link (http://bit.ly/AGSA2025). AGSA is a software tool that is available for free and is used to generate a circular pictogram consisting of twelve segments, each representing one of the twelve aspects of GAC. The color of each segment in the pictogram varies from deep green (indicating the lowest ecological impact) to deep red (indicating the highest ecological impact), depending on how the analysis approach affects the environment, and includes a valuation score in the center of AGSA’s pictogram (83.33) as shown in in Fig. 3b.

#### Carbon footprint

The environment is being disturbed by rising concentrations of greenhouse gases in the atmosphere, which are leading to severe global warming and related effects. Adhering to the principle that only quantifiable data can be controlled, the measurement of Globally, the carbon footprints of various products, bodies, and activities indicate how greenhouse gas-intensive they are^33^. To provide a more holistic view of sustainability, evaluating the carbon footprint of the analytical developed method according to bit.ly/CaFRI with a score of 77 as shown in Fig. 3c. Our HPLC method demonstrated usefulness and applicability.

### Blueness Assessment of the developed methods

#### The blue applicability grade index (BAGI)

**Fig. 4:**
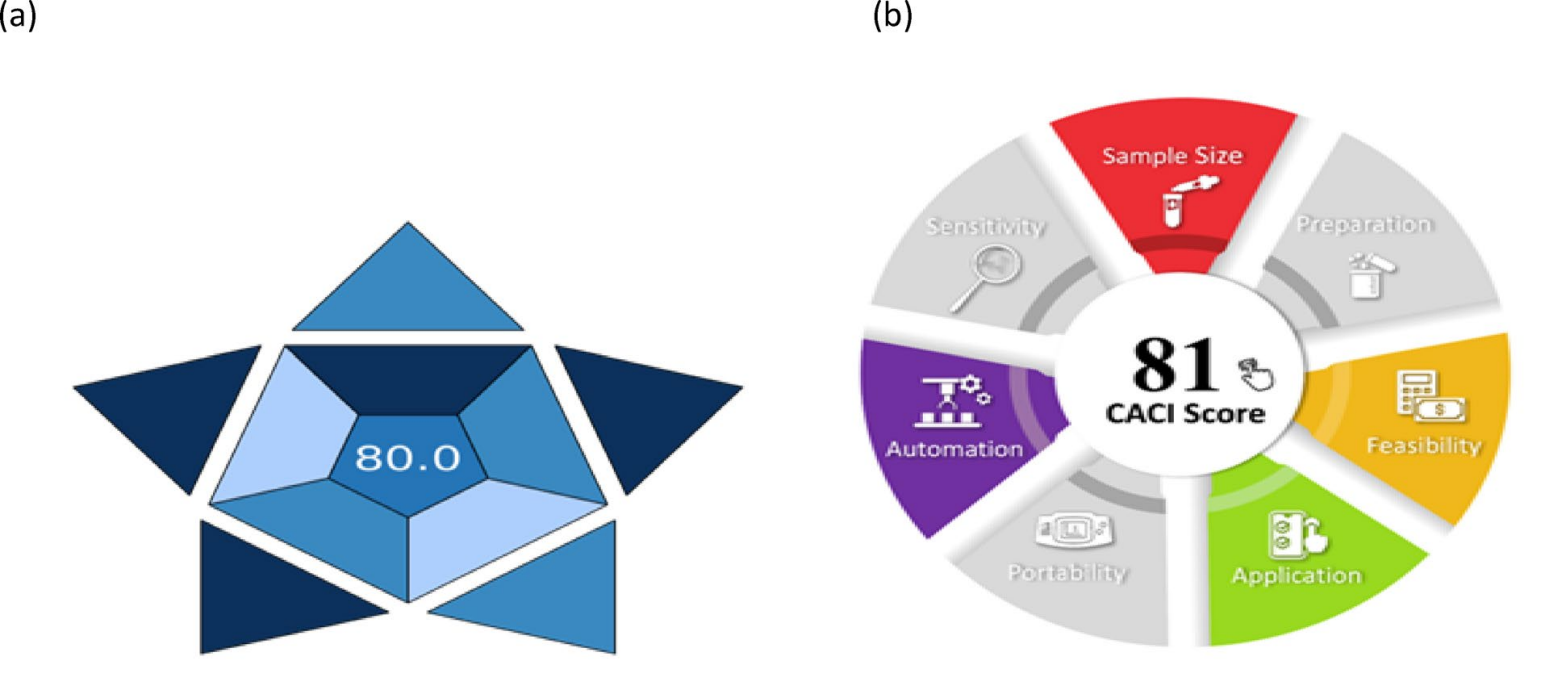
Blueness assessment of the developed method by BAGI (**a**) and CACI (**b**) pictograms.

It assesses the practicality of analytical chemistry methods by assigning scores from 25 to 100. A higher score indicates a more practical method. It enables quick identification of the strengths and weaknesses of a method in terms of its applicability and facilitates comparison of the performance of various analytical methods^34^. The strategy needs to score higher than 60. The nature of analysis, equipment, various compounds, sampling needs, sample size, sample handling capacity, amount of samples processed per hour, automating level, preconcentration needs, and required reagents/materials are all included in BAGI’s validation criteria. Every category is given a color according to these standards, which range from dark blue (signaling high compliance, application, or suitability) to white (signaling non-compliance). With a BAGI score of 80, our HPLC method demonstrated usefulness and applicability (Fig. 4a).

#### Click analytical chemistry index (CACI)

Inspired by the ease of use and dependability of click chemistry, the CACI is based on offering a useful, effective, and intuitive framework for assessing and contrasting analytical techniques. Sample size, preparation, practicality, application, portability, sensitivity, and automation are important factors that are rated to emphasize the method’s conformity to these guidelines. Before the technique can be evaluated by CACI, method validation is a necessary prerequisite. The CACI pictogram’s color code represents the method’s performance in each area. Excellent performance is shown by a colored pictogram score of 81, middling performance is indicated by gray, and black indicates inadequate performance or failure to meet the intended standards, as shown in Fig. 4b. Users may rapidly evaluate the method’s advantages and disadvantages across several factors thanks to this user-friendly framework^35^.

### White analytical chemistry (WAC) of the developed methods

**Fig. 5:**
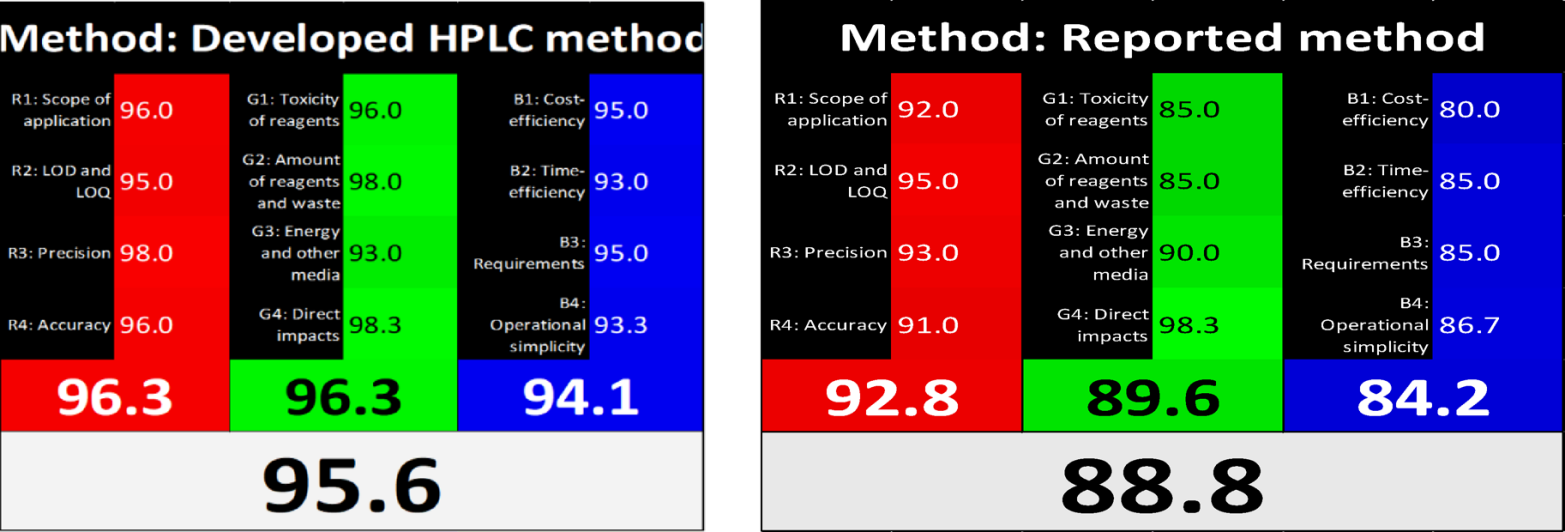
White assessment of the suggested and reported method by WAC pictograms.

It is a relatively new concept for evaluating the cost-effectiveness, environmental friendliness, and efficiency of sample analysis using analytical methods. According to the principles of white analytical chemistry, it is imperative to substitute safer and more cost-effective alternatives for these hazardous organic solvents to safeguard the environment and aquatic life. Red, green, and blue (RGB) are combined to create the color white. To indicate the degree of whiteness accomplished, a numerical score out of 100 is subsequently produced. The percentages of each color are displayed graphically, along with the final result, which should be white. Excessive amounts of each color, especially white, suggest a successful analytical process^19–38^, and we compared with another reported method^18^, but our developed method is better, as shown in Fig. 5.

## Conclusion

A green, selective, accurate, cost-effective, and quantitative Micellar HPLC method was established to separate and assess MET and BISO in the presence of toxic impurities CGD and MLN as co-administration drugs with a linearity range (1.0–25.0 µg/mL) in human plasma with different concentrations. The procedure was effectively validated by ICH criteria. The optimal conditions were achieved using a mobile phase consisting of 0.1 M SDS, 0.1% ortho-phosphoric acid in water, and 10% isopropanol at a flow rate of 1.0 mL/min. The pH was adjusted to 5.0 using triethylamine, and the column temperature was maintained at 25 °C on a symmetry C18 column (Kinetex 1.7µ C18 100A, 2.1-mm × 50-mm), with detection at 230 nm. The greenness of the developed method was evaluated by different greenness assessment metrics, namely, the Analytical eco-scale system which the eco-score value was 81, the modified Green Analytical Procedure Index (MoGAPI) with pictogram score of 89, the Analytical Green Star Area (AGSA) with pictogram score of 83.33, the carbon footprints with pictogram score of 77, the Blue Applicability Grade Index (BAGI) with pictogram score of 80, Click Analytical Chemistry Index (CACI) with pictogram score of 81 and White analytical chemistry (WAC) with pictogram score of 95.6 and compared with another reported method with pictogram score of 88.8. The use of a quantitative analytical green approach helped to guarantee protection from harmful environmental exposure and analyzers, along with the implementation of the Blueness assessment using BAGI tools. This method can be smoothly incorporated into pharmaceutical and medicinal compounds for regular quality control procedures.

## Supplementary Information

Below is the link to the electronic supplementary material.

Supplementary Material 1

## Data Availability

For further information regarding the data used in this study, Please contact: [Mona Abd Elnasser Labib Elsayed], [monaabdelnasser89@gmail.com].
