# Supplementary material for: Eco-friendly micellar HPLC for metformin and bisoprolol analysis in diverse matrices with a green and blueness perspective on drug purity and safety
Source: Sci Rep. 2025 Oct 30;15:37930. doi: 10.1038/s41598-025-22712-w (PMC12575862; doi:10.1038/s41598-025-22712-w)
Supplement: Supplementary file 1 — Supplementary Material 1 [file 41598_2025_22712_MOESM1_ESM.docx]

**Eco-Friendly Micellar HPLC for Metformin and Bisoprolol Analysis in Diverse Matrices: A Green and Blueness Perspective on Drug Purity and Safety**

**Supplementary material:**

**Figures**

**
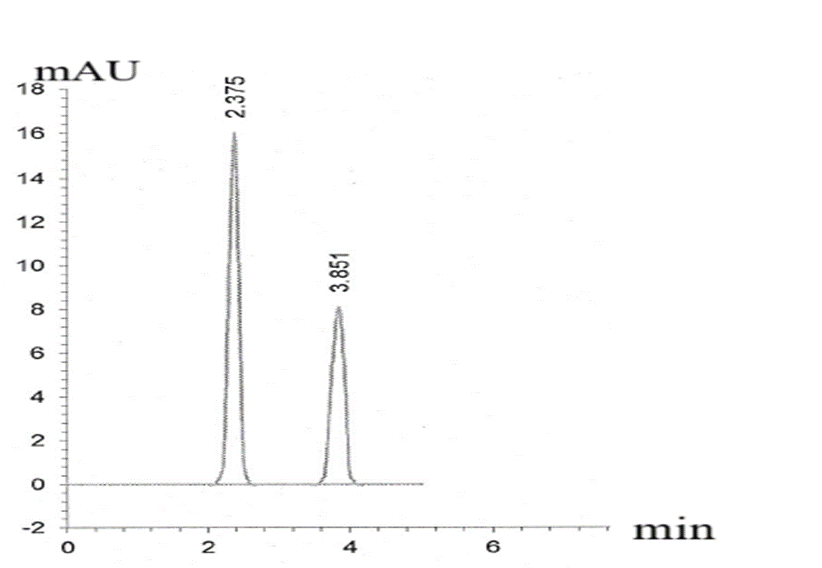
**

**Figure S1:** HPLC chromatogram showing prepared pharmaceutical of MET (10 μg/mL) and BISO (10 μg/mL) with retention time are (2.375) and (3.851) respectively.


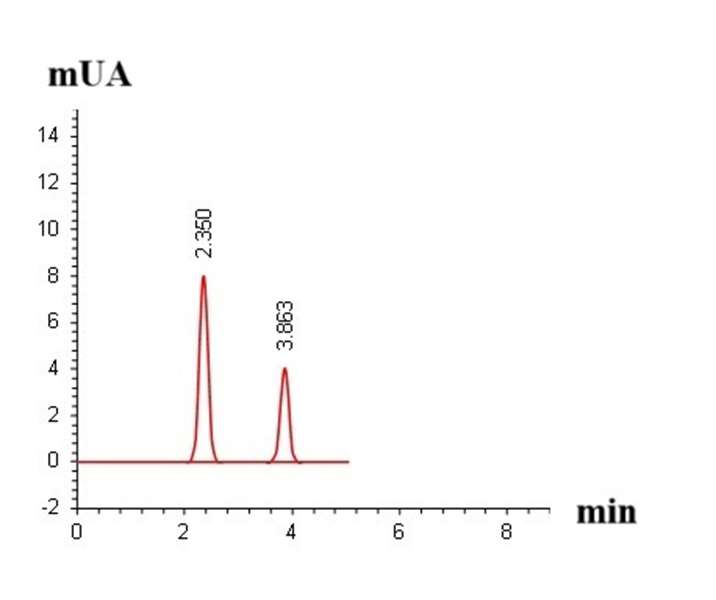


**Figure S2**: HPLC chromatogram showing separation of mixture of (5 μg/mL) of both MET and BISO in their tablet dosage form with retention time are (2.350) and (3.863) respectively.


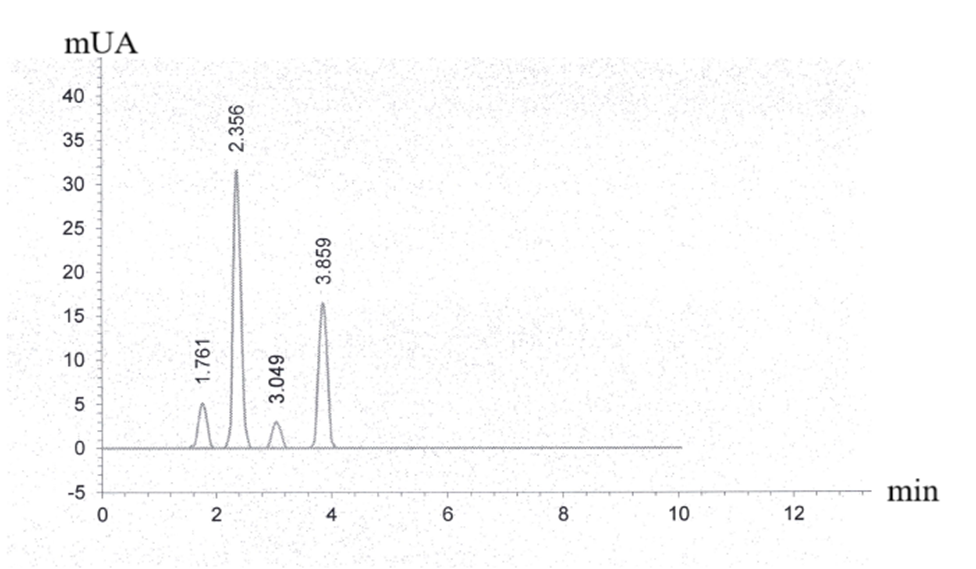


**Figure S3:** HPLC chromatogram of (10 μg/mL) of each CGD, MET, MLN and BISO with retention time (1.761), (2.356), (3.049) and (3.859) respectively.

1.
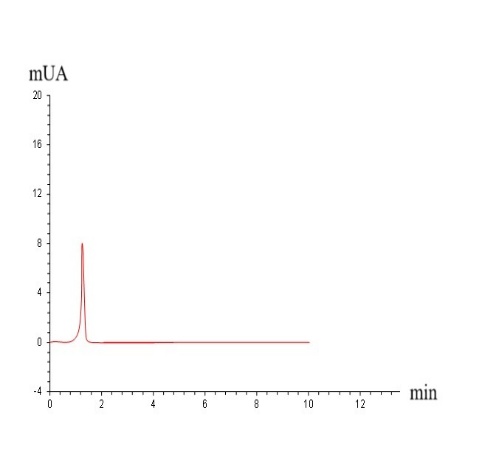
 (b)


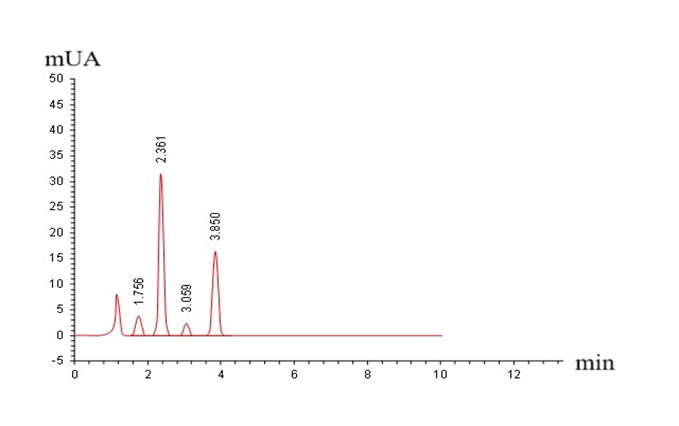


**Figure S4**: Chromatograms of (a) blank plasma; (b) HPLC chromatogram of (10 μg/ml) of each CGD, MTF, MLN and BISO with retention time (1.756), (2.361), (3.059) and (3.850) respectively in spiked plasma.
